# Estimation of Vertical Ground Reaction Forces During Vertical Jumping in Children Using OpenCap

**DOI:** 10.3390/s26113375

**Published:** 2026-05-26

**Authors:** Jiongyi You, Zhicheng Lin, Baifa Zhang

**Affiliations:** School of Sports, Southwest University, Chongqing 400715, China; xiaoyanapollo@gmail.com (J.Y.); dalinsama666@email.swu.edu.cn (Z.L.)

**Keywords:** OpenCap, children, vertical jump, vertical ground reaction force, markerless motion capture

## Abstract

**Highlights:**

**What are the main findings?**

- OpenCap showed moderate-to-high validity for estimating vertical ground reaction force variables during vertical jumping in young children, with strong agreement for phase durations and mean impulse.
- The best estimation performance was observed in the propulsive phase, whereas peak force during the landing stabilization phase was substantially underestimated.

**What are the implications of the main findings?**

- OpenCap may provide a practical and low-cost method for assessing vertical ground reaction force characteristics during children’s vertical jumping outside laboratory settings.
- This approach may support large-scale routine assessment in kindergartens and school physical education, while caution is needed when interpreting landing peak force outcomes.

**Abstract:**

Vertical ground reaction force is an important parameter for describing the developmental characteristics of young children’s vertical jumping. However, its application in large-scale physical fitness monitoring and routine teaching practice is greatly limited. Previous studies have used OpenCap to estimate vertical ground reaction force during adult jumping tasks and have provided preliminary validation, but its effectiveness in young children remains unclear. To examine the correlation and agreement of vertical ground reaction force (GRF) estimated by the OpenCap markerless motion capture system during young children’s vertical jumping and to explore the characteristics of vertical GRF estimated by OpenCap during the vertical jump. Kinematic and kinetic data during vertical jumping were synchronously collected from 16 young children using the OpenCap markerless motion capture system and a three-dimensional force platform, with each child completing three trials. Kinematic data were acquired using the OpenCap markerless motion capture system, and the vertical acceleration of the whole-body center of mass was calculated to estimate vertical GRF based on Newton’s second law. Pearson linear correlation analysis and Bland–Altman analysis were used to examine the differences in characteristics between the estimated vertical GRF and the measured vertical GRF. The vertical GRF characteristics estimated by OpenCap showed moderate-to-high correlations with the measured values. Specifically, the time and mean impulse during the push-off phase, flight phase, and landing stabilization phase were highly correlated (r > 0.85), while the peak force and mean force during the push-off phase showed moderate-to-high correlations (r > 0.7). Bland–Altman analysis showed that the bias in time and impulse during the vertical jump was less than 15%, indicating relatively high agreement; however, the bias in peak force during the landing phase exceeded 40%, indicating weak agreement. These results suggest that the OpenCap markerless motion capture system can effectively estimate vertical GRF characteristics during young children’s vertical jumping, with the best performance observed for vertical GRF variables in the push-off phase. The method used in this study may be applied to obtain vertical GRF during young children’s vertical jumping in non-laboratory settings and to assist in evaluating the developmental level of young children’s vertical jump performance. Nevertheless, OpenCap-derived rapid impact variables, particularly landing peak force, should be interpreted with caution.

## 1. Introduction

Early childhood is a critical period for motor development. Investigating the developmental characteristics of different fundamental movement skills during this stage is essential for helping young children improve fundamental movement skills, master various sports, increase physical activity, and develop lifelong exercise habits [1,2,3]. The Guidelines for Learning and Development of Children Aged 3–6, issued by the Ministry of Education of China (Jiaoji’er [2012] No. 4) [4], likewise emphasize that motor development, as an important component of the health domain in early childhood, is not only a key indicator of children’s physical and mental health but also the foundation for learning and development in other domains. The vertical jump belongs to the locomotor skill category at the base of Seefeldt’s proficiency barrier pyramid model [5], typically begins to develop around the age of two [6], and serves as a foundational skill for complex sport tasks such as basketball, volleyball, and soccer [7]. Delayed development of vertical jumping may hinder children from overcoming the proficiency barrier, preventing them from successfully mastering the more complex sport tasks at the top of the pyramid model and consequently leading to problems in physical fitness and health [8,9].

Vertical ground reaction force (GRF) is an important parameter for characterizing the developmental features of young children’s vertical jumping [10]. At present, three-dimensional force plates are considered the gold standard for measuring vertical GRF; however, their high equipment cost greatly limits their application in large-scale physical fitness monitoring and routine teaching practice [11]. With the development of computer vision and deep learning technologies, markerless motion capture has gradually emerged as a low-cost and convenient alternative to force plates by obtaining kinematic parameters of body segments and estimating vertical GRF through summing inertial forces based on segmental center-of-mass accelerations and Newton’s second law [12,13]. OpenCap, a system recently developed by Stanford University [14], uses multi-view smartphone videos and integrates pose estimation algorithms, deep learning models, and musculoskeletal dynamic simulation to estimate biomechanical parameters in outdoor settings. With advantages such as low cost, flexible deployment, and minimal site requirements, it provides a feasible solution for tracking training effects and monitoring movement quality in school physical education and grassroots training settings. Recent studies have applied OpenCap to several movement tasks, including gait, lower-limb functional movements, and cycling, suggesting its potential for practical biomechanical assessment outside traditional laboratories [15,16,17,18]. In jumping tasks, OpenCap has been preliminarily validated for estimating vertical GRF during adult vertical jumping [19]. However, studies on the application of OpenCap to young children’s vertical jumping remain limited. Children differ substantially from adults in body proportions, segmental mass distribution, skeletal maturity, and neuromuscular control. These differences may affect the accuracy of whole-body center-of-mass estimation and, consequently, the derivation of vertical GRF from kinematic data [20,21,22]. Moreover, systematic evidence is still lacking as to whether the kinematic data it acquires can reliably support vertical GRF estimation. Therefore, this study aims to validate the feasibility and accuracy of estimating vertical GRF in young children’s vertical jump based on kinematic data collected by OpenCap.

## 2. Methods

### 2.1. Participants

Sixteen preschool children (eight males and eight females; age = 5.99 ± 0.25 years, body mass = 21.87 ± 2.95 kg, body height = 1.18 ± 0.05 m) were recruited from a kindergarten in Beibei District, Chongqing, China, and participated in this study. Participant characteristics are shown in Table 1. Children who were physically healthy and had normal cognitive and motor development were included. Children were excluded if they had previously sustained a lower-limb injury (e.g., fracture or severe sprain), had a diagnosed developmental disorder, or had any reported disease that might affect lower-limb movement.

### 2.2. Data Collection

**Figure 1:**
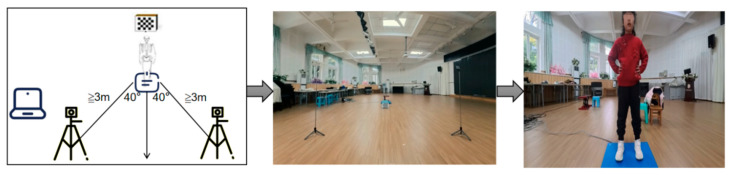
Schematic diagram of the experimental setup.

As illustrated in Figure 1, vertical jump data were collected synchronously using the OpenCap markerless motion capture system and a force platform. Two iOS devices (iPhone 12, Apple Inc., Cupertino, CA, USA) were used for motion capture through the OpenCap application (Model Health, Inc., Stanford University, USA), with a sampling frequency of 240 Hz. Before testing, the two iOS devices were mounted on two tripods with a height of 1 m. Each device was positioned approximately 4 m in front of the participant, with a distance of 3 m between the two cameras and an angle of 40° between them. An A4-printed checkerboard (210 × 175 mm) was used for spatial calibration. Participants were instructed to stand in a relaxed posture facing the iOS devices, with their feet shoulder-width apart, arms resting alongside the body, and palms facing outward [14]. After calibration, OpenCap recorded the video data at 240 Hz, and the musculoskeletal model was scaled to match each child’s anthropometric measurements.

A Kistler force platform (9260AA, Kistler, Winterthur, Switzerland) was used to record vertical GRF data at a sampling frequency of 1000 Hz. Prior to formal testing, all participants wore tight-fitting clothing and completed a brief familiarization session. All participants performed the vertical jump test barefoot. Given the limited motor control and task comprehension capacities typical of children under 6 years of age, the experimenter first demonstrated the vertical jump task and provided age-appropriate verbal instructions. Participants were instructed to stand at the center of the force platform with both hands placed on their hips and feet positioned shoulder-width apart. Upon receiving the start command, they were asked to jump vertically in place, land with both feet on the force platform, and remain stationary after landing. Each participant subsequently completed two to three practice trials. Formal data collection commenced only when the participant demonstrated adequate understanding of the instructions and consistent task performance. During formal testing, participants first stood quietly on the force platform for 3 s. Once the OpenCap system was activated, the experimenter issued a verbal start command, whereupon participants performed a single vertical jump in place. Following landing, participants remained stationary on the force platform for an additional 3 s before stepping off. A valid trial was defined as one in which the child successfully took off and landed on the force platform. Each participant completed three valid trials, with at least 1 min of rest between trials to minimize the influence of fatigue on jump performance. The experiment was conducted indoors in a spacious environment with stable lighting conditions, and natural light was used to reduce external interference.

### 2.3. Data Analysis

The vertical jump was divided into four key events: the propulsion onset, defined as the instant after the end of the downward movement and before take-off when the vertical GRF first exceeded body weight; the take-off instant, defined as the instant after the end of propulsion when the vertical GRF decreased and remained below 5% of body weight; the landing instant, defined as the instant after the end of flight when the vertical GRF increased again and remained above 5% of body weight; and the stabilization instant, defined as the last instant at which the vertical GRF fell below 5% of body weight and remained so for at least 0.1 s [23]. Based on these four events, the jump was further divided into three phases: the propulsive phase (from propulsion onset to take-off), the flight phase (from take-off to landing), and the landing stabilization phase (from landing to stabilization).

For OpenCap-based GRF estimation, the vertical jump was treated as a primarily vertical movement task. It was assumed that the dominant external force, apart from gravity, was the vertical GRF generated at the foot-ground interface, with other external forces such as air resistance considered negligible. This study referred to the mechanics-based GRF estimation approach proposed by Verheul et al., in which the vertical ground reaction force is derived from human kinematic data according to Newton’s second law. However, the method used in the present study differed from that of Verheul et al. [19]. Specifically, Verheul et al. estimated vertical GRF by calculating the vertical acceleration of 22 body segments and summing the products of each segment’s mass and vertical acceleration. In the present study, vertical GRF during vertical jumping was estimated from whole-body kinematic data by calculating the acceleration of the whole-body center of mass and applying Newton’s second law. Data processing was performed using the OpenCap-processing library workflow in combination with a full-body musculoskeletal model implemented in OpenSim. The full-body musculoskeletal model consisted of 22 body segments, each defined by its segmental mass properties and corresponding local center-of-mass position.

(1) At any time point t, the position of the whole-body center of mass (CoM) was calculated as the mass-weighted average of the center-of-mass positions of all body segments, where m_i_ and r_i_(t) denote the mass and center-of-mass position of the ith segment, respectively, and N is the total number of body segments.

(1) $$r_{COM}\left(t\right)=\frac{1}{\sum_{i=1}^{N}m_{i}}\sum_{i=1}^{N}m_{i}r_{i}(t)$$

(2) The vertical acceleration was obtained by differentiating the vertical displacement of the whole-body CoM with respect to time.

(2) $$a_{COM}\left(t\right)=\frac{∆r_{COM(t)}}{∆t^{2}}$$

(3) On this basis, according to Newton’s second law, the vertical GRF was derived from the vertical acceleration of the whole-body CoM, where m is the participant’s total body mass and g is the gravitational acceleration (9.81 m·s^−2^).

(3) $$F_{GRF}=m(a_{COM}+g)$$

(4) To facilitate comparisons among different participants, the estimated vertical GRF was normalized and expressed in multiples of body weight (BW).

(4) $${vGRF}_{BW}=\frac{a_{COM}+g}{g}$$

After estimating the vertical GRF, the resulting kinetic data were further processed in Python 3.13. First, following the approach of Verheul et al., the estimated vertical GRF data were filtered using a second-order low-pass Butterworth filter with a cutoff frequency of 4 Hz [19]. This filtering procedure was primarily applied to reduce baseline noise introduced by errors in upper body segment motion estimation, thereby improving the stability of the vertical GRF profiles derived from segmental accelerations. Subsequently, to unify the time scale and facilitate comparison with the force plate data, the filtered vertical GRF signal was resampled to 1000 Hz using linear interpolation [24].

### 2.4. Statistics

Prior to statistical analyses, vertical GRF profiles and OpenCap motion capture outputs were inspected to identify potential outliers and inconsistent trials. Trials were excluded only when the estimated GRF profiles were unanalyzable due to poor motion capture quality, such as physiologically implausible segment orientations or incorrect landing on the force platform. No trials met these exclusion criteria. The durations of the push-off, flight, and landing stabilization phases, together with the peak force, mean force, and impulse during the push-off and landing phases, were selected for comparison between OpenCap and the force platform. Statistical analysis was performed using SPSS 27.0. Data normality was assessed using the Shapiro–Wilk test, and no significant departures from normality were detected (*p* > 0.05). All variables are expressed as mean ± standard deviation. Descriptive statistics were used to present the means and standard deviations of the data. Pearson correlation analysis was used to assess the correlation between the force-platform measurements and the OpenCap-estimated values, and statistical significance was set at *p* < 0.05. Relative estimation bias was calculated and plotted using MATLAB (version R2024b, MathWorks, Natick, MA, USA) to evaluate the agreement between the measured and estimated values. Bland–Altman plots with 95% limits of agreement (LoA) were generated to evaluate systematic bias and the range of agreement between trials. The smaller the absolute value of the relative estimation bias, the better the agreement between the force-platform measurements and the OpenCap estimations. Reliability was assessed using the intraclass correlation coefficient (ICC (2,1)). To quantify the discrepancy between the two methods, the root mean square error (RMSE) and mean absolute error (MAE) were also calculated. RMSE reflects the overall error magnitude and is more sensitive to large deviations, whereas MAE represents the average absolute difference between methods [18,25].

## 3. Results

**Figure 2:**
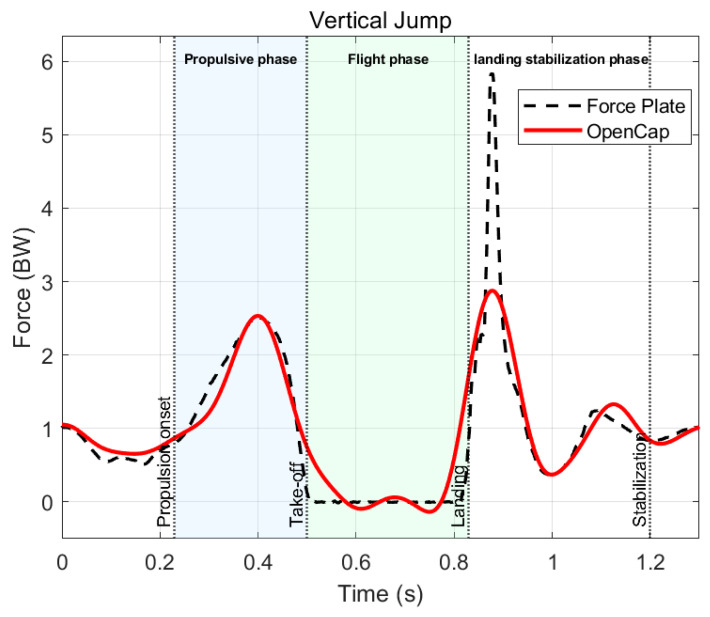
Representative measured (black dashed line) and estimated (red solid line) ground reaction forces during the vertical jump.

A total of 48 vertical jump trials were collected and analyzed. Under the guidance of the researchers, all participants successfully completed the prescribed vertical jump task. Figure 2 shows representative vertical GRF curves measured by the force platform and estimated by OpenCap during the vertical jump.

Table 2 presents the means, standard deviations, Pearson correlation coefficients, RMSE, MAE, and ICC (model 2,1) for the vertical GRF characteristics derived from force-platform measurements and OpenCap estimations during the different phases of children’s vertical jumping. The results indicated that the OpenCap-estimated vertical GRF variables were highly correlated with the measured values for phase duration (r = 0.90–0.94), impulse (r = 0.83–0.98), and mean force (r = 0.82), with high ICC values (0.82–0.97) and relatively small RMSE (0.04–0.10) and MAE (0.03–0.09). By contrast, larger discrepancies were observed for peak force variables, which showed low-to-moderate ICC values (0.29–0.75). Although peak force during the push-off phase showed a relatively high correlation (r = 0.70), the correlation for peak force during the landing stabilization phase was relatively low (r = 0.53).

**Figure 3:**
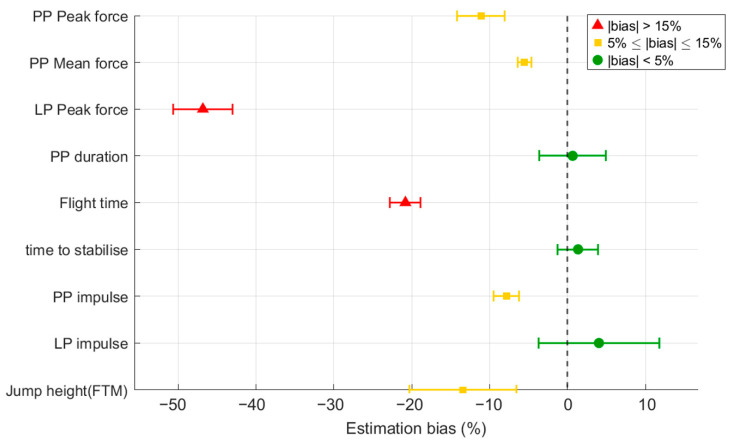
Percentage bias and limits of agreement between the measured and estimated vertical ground reaction force variables for the vertical jump. Circles/green, squares/amber, and triangles/red represent a bias/limit of agreement of <5%, 5–15%, or >15%. LP = landing phase, PP = propulsive phase, FTM = flight time method.

**Figure 4:**
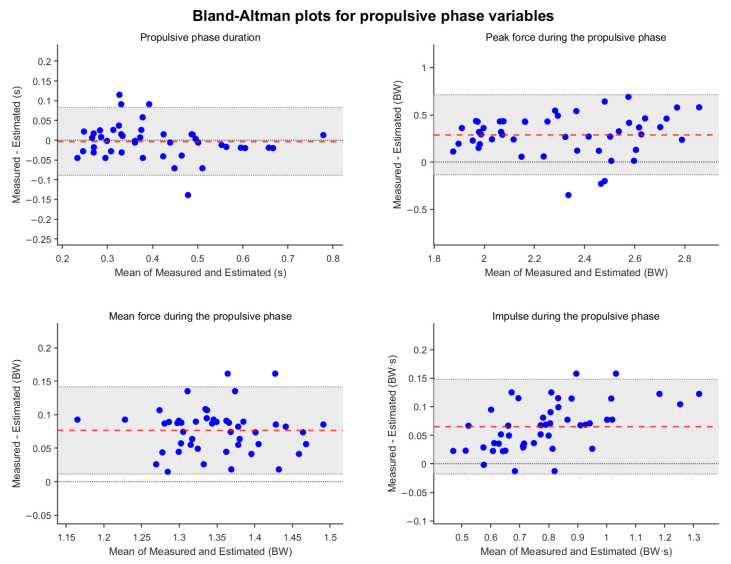

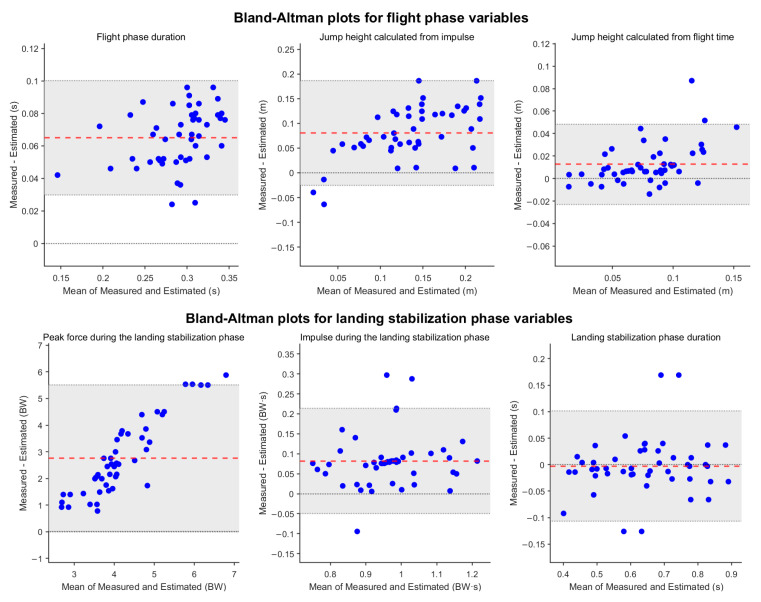
Bland–Altman plots showing the limits of agreement between force platform measurements and OpenCap estimations for vertical GRF variables during the vertical jump. Each subplot displays the difference between the two methods (y-axis) against the mean of the two methods (x-axis) for a specific variable. The red dashed line indicates the mean bias, and the gray shaded area represents the 95% limits of agreement (LoA), with the dotted lines indicating the upper and lower boundaries.

Figure 3 and Figure 4 show the Bland–Altman analysis of the vertical GRF characteristics derived from force platform measurements and OpenCap estimations across different phases of children’s vertical jumping. The results showed that the biases for push-off time, landing stabilization time, and impulse during the landing stabilization phase were within ±5%, indicating good agreement. The biases for peak force, mean force, and impulse during the propulsive phase ranged from −5% to −15%, suggesting that OpenCap slightly underestimated these variables, although the overall agreement remained acceptable. In contrast, the bias for peak force during the landing stabilization phase exceeded 40% and increased with the mean value, indicating that OpenCap substantially underestimated landing peak force. Overall, OpenCap demonstrated good agreement for temporal and impulse-related variables, whereas agreement was poorer for peak force variables, particularly during the landing stabilization phase.

## 4. Discussion

This study used the OpenCap markerless motion capture system to obtain whole-body kinematic data and estimated vertical GRF from whole-body center-of-mass acceleration according to Newton’s second law. Simultaneously, a force platform was used to analyze the vertical GRF during young children’s vertical jumping so as to verify the correlation between the estimated and measured values and evaluate the practical value of this method.

The results of the correlation analysis indicated that the phase durations and mean impulses of vertical GRF estimated using the OpenCap markerless motion capture system were highly correlated with the corresponding force-platform measurements (r = 0.83–0.98). This finding is consistent with the results reported by Verheul et al. [19], who estimated phase-specific vertical GRF variables during adult vertical jumping based on OpenCap data (r = 0.90–0.99). The RMSE, MAE, and ICC analyses further indicated that OpenCap estimation accuracy varied across vertical GRF variables in different phases of children’s vertical jumping. Overall, temporal and impulse variables exhibited smaller errors and higher agreement. Specifically, the RMSE, MAE, and ICC values for phase duration were 0.04–0.07 s, 0.03–0.07 s, and 0.91–0.94, respectively. The ICC values for impulse variables ranged from 0.82 to 0.97, indicating that OpenCap provided reliable estimations for variables reflecting the overall characteristics of each movement phase. Mean force and peak force during the propulsive phase also demonstrated moderate to good agreement, with ICC values of 0.88 and 0.75, respectively. However, peak force during the landing stabilization phase exhibited the largest error (RMSE = 3.09 BW, MAE = 2.76 BW), with an ICC of only 0.29. The agreement analysis revealed that, among the estimated vertical GRF variables, the bias values for the push-off phase duration, landing cushioning phase duration, and mean impulse were all below 5%, indicating high agreement. The bias values for the peak force and mean force during the push-off phase were between 5% and 15%. Previous studies have generally considered a bias of less than 15% to indicate acceptable estimation accuracy for a given variable [19,26,27]. Therefore, the present findings suggest that OpenCap can provide relatively accurate estimates for certain vertical GRF characteristics during vertical jumping in young children. However, the correlation coefficient for the estimated peak force during the landing phase was relatively low (r = 0.53), with a bias exceeding 40%. OpenCap-estimated values showed systematic underestimation during the landing phase relative to force platform measurements. This discrepancy may be attributable to the difference in sampling rates: the force platform acquired data at 1000 Hz, whereas OpenCap kinematic data were collected at 240 Hz. Peak force at initial contact occurs within a very brief time window, during which velocity and force change rapidly; this transient response may be difficult for OpenCap to capture accurately [19]. Furthermore, filtering of the video-derived center-of-mass acceleration may have additionally attenuated high-frequency impact components. These findings suggest that the OpenCap markerless motion capture system is more suitable for estimating push-off phase variables in children’s vertical jumps, whereas landing peak force estimates should be interpreted with caution.

Vertical jumping requires a certain level of lower-limb strength and dynamic balance in children and is therefore commonly used as an auxiliary indicator for evaluating the development of coordination and leg strength during early childhood [28]. Most children have acquired this skill by approximately two years of age, and a relatively mature movement pattern typically emerges by around five years of age. Previous studies have demonstrated a close relationship between vertical GRF during the push-off phase and vertical jump performance. In a vertical jump experiment with different countermovement depths, Kirby et al. [29] examined the relationships among peak force, mean impulse during the push-off phase, and jump height, and found that push-off impulse was positively correlated with jump height, whereas the correlation between peak force and jump height was relatively weak. Barker et al. [30] investigated the changes in vertical GRF across the different phases of the vertical jump and reported that vertical GRF power during the push-off phase was significantly associated with jump height. These findings indicate that the push-off phase is a key phase in determining vertical jump height. Research has further demonstrated that, as movement patterns become more mature, the vertical GRF characteristics during the push-off phase of children’s vertical jumping show continuous improvement. By comparing the vertical GRF characteristics of children with different levels of movement maturity during vertical jumping, Meylan et al. [31] found that both the mean and peak vertical GRF power during the push-off phase increased with improvements in movement maturity [31,32]. Floría et al. [10] compared vertical GRF differences during vertical jumping among girls with different developmental levels and likewise found that the higher-level group exhibited greater vertical GRF during the initial push-off phase than the lower-level group. Therefore, the estimation of vertical GRF characteristics during vertical jumping based on OpenCap may provide an objective approach for assisting in the assessment of the developmental level of young children’s vertical jump performance. Meanwhile, compared with professional equipment such as force platforms, OpenCap offers advantages including lower cost and greater ease of use [14], which supports its potential use in standardized movement assessments in kindergartens and school physical education settings.

This study has several limitations. First, the relatively large discrepancies observed for landing peak force may have compromised the accuracy of impact-related outcomes. Accordingly, findings involving landing peak force should be interpreted with caution. Second, only vertical jumping in young children was examined. Consequently, the performance of OpenCap across different jump intensities, amplitudes, and movement patterns remains unclear. Future studies should incorporate tasks such as low-amplitude jumps, maximal-effort jumps, drop jumps, and progressively loaded jumps to determine whether estimation errors vary with movement intensity and impact characteristics. Third, non-jumping tasks were not included. Tasks involving smoother acceleration profiles, such as walking, squatting, and sit-to-stand movements, may be more amenable to kinematics-based GRF estimation. Future studies should therefore examine these movements to identify the conditions under which OpenCap yields reliable estimates. Fourth, this study was limited to young children, and estimation errors were not compared across age groups, BMI categories, or body morphologies. Future research should recruit participants varying in age and body composition to determine whether systematic biases exist within the OpenCap estimation pipeline. Fifth, although 48 valid jump trials were collected, these were obtained from 16 children with three repeated trials per participant. This repeated-measures design improved the stability of within-participant estimates but resulted in non-independent observations. Accordingly, the small sample size and within-subject dependence should be considered when interpreting these findings. Future studies employing larger samples and statistical approaches that account for repeated measurements are needed to confirm these results.

## 5. Conclusions

This study demonstrates that combining OpenCap markerless motion capture with vertical GRF estimation derived from whole-body center-of-mass acceleration via Newton’s second law can effectively capture selected vertical GRF characteristics during vertical jumping in young children. This approach offers a practical, low-cost method for acquiring vertical GRF data in non-laboratory settings, supporting its potential application in kindergarten and school-based movement assessments under standardized testing conditions. Nevertheless, given that OpenCap systematically underestimated landing peak force, variables related to rapid impact derived from OpenCap-based GRF estimates should be interpreted with caution.

## Figures and Tables

**Table 1:** Participant characteristics.

N	Sex (Male/Female)	Age (Years)	Height (m)	Body Mass (kg)	BMI (kg/m^2^)
16	8/8	5.99 ± 0.25	1.18 ± 0.05	21.87 ± 2.95	15.77 ± 1.27

**Table 2:** Descriptive statistics, Pearson correlation coefficients, RMSE, MAE, and ICC for measured and estimated ground reaction force variables during the vertical jump.

Variable	Measured	Estimated	Difference	r	RMSE	MAE	ICC (2,1)
Propulsive phase duration (s)	0.39 ± 0.12	0.39 ± 0.13	0.00 ± 0.05	0.94	0.04	0.03	0.94
Peak force during the propulsive phase (BW)	2.44 ± 0.31	2.18 ± 0.31	0.26 ± 0.24	0.70	0.36	0.32	0.75
Mean force during the propulsive phase (BW)	1.36 ± 0.06	1.28 ± 0.06	0.08 ± 0.03	0.82	0.08	0.08	0.88
Mean impulse during the propulsive phase (BW·s)	0.78 ± 0.19	0.71 ± 0.17	0.06 ± 0.04	0.98	0.08	0.07	0.97
Flight phase duration (s)	0.31 ± 0.04	0.25 ± 0.04	0.06 ± 0.02	0.90	0.07	0.07	0.91
Jump height calculated from impulse (m)	0.16 ± 0.08	0.08 ± 0.03	0.08 ± 0.06	0.63	0.10	0.09	0.59
Jump height calculated from flight time (m)	0.09 ± 0.04	0.07 ± 0.03	0.02 ± 0.02	0.87	0.02	0.02	0.84
Peak force during the landing stabilization phase (BW)	5.52 ± 1.34	2.80 ± 0.43	2.76 ± 1.34	0.53	3.09	2.76	0.29
Mean impulse during the landing stabilization phase (BW·s)	1.02 ± 0.12	0.94 ± 0.13	0.09 ± 0.07	0.83	0.11	0.09	0.82
Landing stabilization phase duration (s)	0.60 ± 0.12	0.61 ± 0.12	0.00 ± 0.05	0.93	0.05	0.03	0.92

Note: BW = body weight; RMSE = root mean square error; MAE = mean absolute error; ICC = intraclass correlation coefficient.

## Data Availability

The datasets generated and/or analyzed during the current study are available from the corresponding author upon reasonable request.
